# Utility of S100A12 as an Early Biomarker in Patients With ST-Segment Elevation Myocardial Infarction

**DOI:** 10.3389/fcvm.2021.747511

**Published:** 2021-12-17

**Authors:** Xiaolin Zhang, Minghui Cheng, Naijing Gao, Yi Li, Chenghui Yan, Xiaoxiang Tian, Dan Liu, Miaohan Qiu, Xiaozeng Wang, Bo Luan, Jie Deng, Shouli Wang, Hongyan Tian, Geng Wang, Xinliang Ma, Gregg W. Stone, Yaling Han

**Affiliations:** ^1^Cardiovascular Research Institute and Department of Cardiology, The General Hospital of Northern Theater Command, Shenyang, China; ^2^Department of Cardiology, Liaoning Provincial People's Hospital, Shenyang, China; ^3^Department of Cardiology, Second Affiliated Hospital of Xi'an Jiaotong University, Xi'an, China; ^4^Department of Cardiology, General Hospital of the Strategic Support Force of the Chinese People's Liberation Army, Beijing, China; ^5^Department of Cardiology First Affiliated Hospital of Xi'an Jiaotong University, Xi'an, China; ^6^Department of Emergency Medicine, Thomas Jefferson University, Philadelphia, PA, United States; ^7^Icahn School of Medicine at Mount Sinai, Mount Sinai Heart and the Cardiovascular Research Foundation, New York, NY, United States

**Keywords:** S100A12, ST-segment elevation myocardial infarction, diagnosis, prognosis, cardiovascular disease(s)

## Abstract

**Importance:** S100A12 is a calcium binding protein which is involved in inflammation and progression of atherosclerosis.

**Objective:** We sought to investigate the utility of S100A12 as a biomarker for the early diagnosis and prognostication of patients presenting with ST-segment elevation myocardial infarction (STEMI).

**Design, Setting, and Participants:** S100A12 was measured in 1023 patients presenting to the emergency department with acute chest pain between June 2012 and November 2015. An independent cohort of 398 patients enrolled at 3 different hospitals served as a validation cohort.

**Main Outcomes and Measures:** The primary clinical endpoint of interest was major adverse cardiac and cerebral events (MACCE) defined as a composite of all-cause death, MI, stroke, or hospitalization for heart failure.

**Results:** A total of 438/1023 patients (42.8%) in the diagnosis cohort were adjudicated as STEMI, among whom plasma S100A12 levels increased within 30 min and peaked 1–2 h after symptom onset. Compared with high-sensitivity cardiac troponin T and creatine kinase-MB isoenzyme, S100A12 more accurately identified STEMI, especially within the first 2 h after symptom onset (area under the curve 0.963 compared with 0.860 for hscTnT and 0.711 for CK-MB, both *P* < 0.05). These results were consistent in the 243-patient validation cohort. The 1-year rate of MACCE was greatest in patients in the highest peak S100A12 tertile, intermediate in the middle tertile and least in the lowest tertile (9.3 vs. 5.7 vs. 3.0% respectively, P_trend_ = 0.0006). By multivariable analysis the peak plasma concentration of S100A12 was an independent predictor of MACCE within 1 year after STEMI (HR, 1.001, 95%CI, 1.000–1.002; *P* = 0.0104).

**Conclusions and Relevance:** S100A12 rapidly identified patients with STEMI, more accurately than other cardiac biomarkers, especially within the first 2 h after symptom onset. The peak plasma S100A12 level was a strong predictor of 1-year prognosis after STEMI.

## Key Points

- Question: Can circulating S100A12 serve as a useful biomarker for the early diagnosis and prognostication of patients with STEMI?
- Findings: Among 1,023 patients (438 of whom had STEMI), S100A12 diagnosed STEMI more accurately than other commonly used biomarkers of myocardial injury (troponin and CK-MB). S100A12 was particularly useful to discriminate the etiology of chest pain within 2 h of symptom onset, before other biomarkers typically rise. Peak S100A12 levels were an independent predictor of MACE within 1 year after STEMI.
- Meaning: S100A12 is a novel diagnostic biomarker which may be used to rapidly identify patients with STEMI, especially in the early hours after symptom onset. The peak plasma concentration of S100A12 also provides prognostic utility within 1 year after STEMI.

## Introduction

Acute myocardial infarction (AMI) is a major worldwide cause of morbidity and mortality (1). Early recognition and intervention in patients with AMI, especially those with ST-segment elevation myocardial infarction (STEMI), is crucial to salvage myocardium and improve long-term prognosis. Currently, diagnosis of STEMI is dependent on early elevation of cardiac biomarkers, most commonly high-sensitivity cardiac troponin T (hscTnT) or creatine kinase MB isoenzyme (CK-MB), in concert with consistent symptoms and an abnormal electrocardiogram (ECG). Optimal treatment of STEMI requires its rapid diagnosis for emergent cardiac catheterization and primary percutaneous coronary intervention. However, patients often present with a non-diagnostic ECG or atypical symptoms (2). In such patients the rapid diagnosis of STEMI (within 2–3 h of symptom onset, the time period in which rapid reperfusion is most beneficial), can be challenging given a delay in rise of biomarkers.

S100A12 is a member of the S100 multigene family, a calcium binding protein which is highly associated with chronic inflammatory disorders, including atherosclerosis and coronary artery disease (CAD) (3, 4). Studies have demonstrated that the expression of S100A12 is up-regulated in coronary artery plaques of patients with sudden cardiac death (5) and carotid artery plaques of patients with transient ischemic attack (6), suggesting a relationship between S100A12 and atherothrombotic events (7). S100A12 predominantly localizes in activated macrophages in atherosclerotic plaques. Circulating S100A12 levels are not elevated in patients with stable coronary artery disease but are increased in those with acute coronary syndromes (ACS) and after percutaneous coronary intervention (PCI) due to acute release of S100A12 from macrophages after plaque rupture or mechanical injury (8, 9). We hypothesize that the plasma concentration of S100A12 might be elevated in the early stage of STEMI due to plaque rupture or erosion, and thus serve as an effective biomarker for the early diagnosis of STEMI in patients with chest pain. In the present study we therefore sought to determine the utility of S100A12 for the early diagnosis and prognostication of STEMI among patients with chest pain.

## Methods

### Study Design and Participants

The present study prospectively enrolled three patient cohorts. The first cohort comprised 1,023 patients presenting with acute chest pain to the emergency department of General Hospital of Northern Theater Command, Shenyang Liaoning Province, China, between June 2012 and November 2015. Patients were included if they presented with acute chest pain and/or discomfort possibly indicating AMI with symptom onset within 24 h, were older than 18 years and were willing to participate in the study. Exclusion criteria included patients with known active inflammatory or autoimmune diseases, severe heart failure, hemodynamic instability, suspected myocarditis or pericarditis, diseases of the hematopoietic system, known severe kidney or liver disease, known malignancy, use of immunosuppressant agents, and previous coronary artery bypass graft surgery. These 1023 patients served as the diagnosis cohort. A second patient group, serving as a validation cohort, comprised 398 patients with the same inclusion and exclusion criteria presenting to the emergency department of three different hospitals (eAppendix 1) between May 2016 and November 2016, The third cohort comprised the subgroup of patients with confirmed STEMI from the first cohort (*n* = 438) and 562 newly enrolled consecutive STEMI patients from the same hospital between January 2016 and May 2017, in which the long-term prognostic value of S100A12 in patients with STEMI was evaluated. This study was approved by ethics committee of all participating centers and all patients signed informed consent.

### Clinical Adjudication

Three independent cardiologists reviewed all available medical records. AMI was defined in accordance with current guidelines and required a typical rise and fall in cardiac biomarkers (troponins and/or CK-MB) with ECG evolution (10). STEMI was differentiated from NSTEMI by the presence of ST-segment elevation in at least two contiguous leads or true posterior infarction with anterior ST-segment depression. Unstable angina pectoris (UAP) was diagnosed in patients with normal biomarker levels and typical angina at rest or rapid progression of previously stable angina with a consistent ECG or positive exercise test or cardiac catheterization showing coronary stenosis of ≥70%. Non-coronary syndromes such as pulmonary thromboembolism (PTE) and aortic dissection (AD) were diagnosed according to current standards (11, 12). Patients with other diagnoses or chest pain of unknown origin were classified as “others.”

### Sample Collection

Blood samples were obtained at the time of arrival at the emergency department for all patients. Depending on the time from symptom onset to hospital arrival, subsequent samples were drawn at 2, 4, 6, 12, and 24 h and at 3, 7, and 30 days after chest pain onset. Sequential sampling was terminated once the diagnosis of STEMI was excluded.

### Outcomes

All confirmed STEMI patients in the third cohort were followed via telephone or outpatient clinic visits at 6 months and 1 year after discharge. The primary outcome was major adverse cardiac and cerebral events (MACCE) at 1 year, a composite of all-cause death, repeat myocardial infarction (MI), stroke, or hospitalization for heart failure. Major secondary outcomes were the individual components of MACCE. MI was defined according to the Third Universal Definition of Myocardial Infarction (10). Stroke was defined as acute focal dysfunction of the brain, retina, or spinal cord lasting longer than 24 h, or of any duration if focal infarction or hemorrhage was confirmed by neuro-imaging (13). All clinical events were adjudicated by physicians blinded to the biomarker analysis results.

### Statistical Analysis

The sample size of the first and second cohorts followed guidance for clinical studies of *in-vitro* diagnostic reagents from the China Food and Drug Administration. For the third cohort, from prior studies we assumed the MACCE rate at 1-year after STEMI would be 7.5%. To determine a 95% confidence interval between 6.0 and 9.0%, with two-sided alpha of 0.05 and power of 90%, and considering 10% loss of follow up, a total of 1,000 patients would be needed, providing 60–90 events. Categorical variables are reported as counts and percentages, and between-group differences were assessed with chi-square or Fisher's exact test. Continuous variables are presented as the mean ± SD and were compared with a two-sample *t*-test or one-way analysis of variance. Unless otherwise specified, a two-sided *P* < 0.05 was considered to indicate statistical significance. Time-to-event data with event rates estimated with the Kaplan-Meier method were compared using the log-rank test. Logistic regression analysis was used to identify the independent predictors of MACCE. Discrimination was quantified by the area under the receiver-operating characteristics curve (ROC AUC) for each biomarker. Statistical analysis was performed using SPSS version 21.0 software (IBM SPSS Inc., Chicago, IL, USA).

## Results

**Figure 1:**
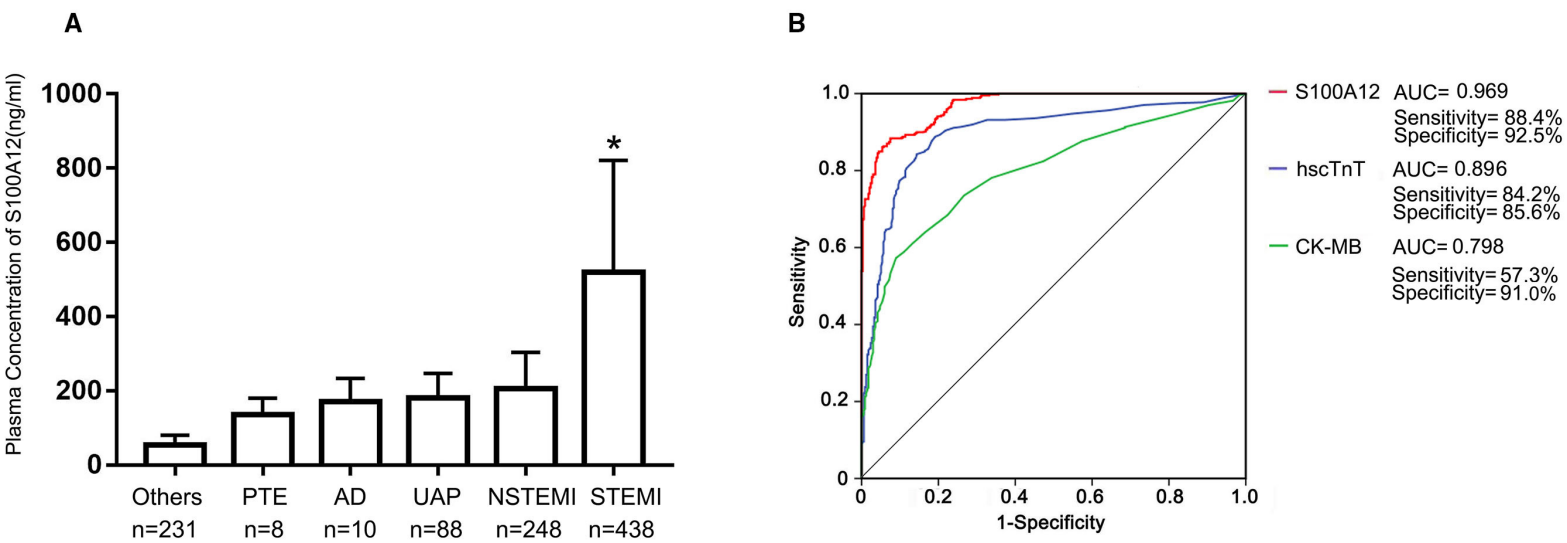
Plasma S100A12 levels and diagnostic accuracy. Baseline plasma S100A12 concentrations at the time of hospital arrival to the emergency department were measured by ELISA in the diagnosis cohort. **(A)** Bars and limit lines represent the mean ± SD. UAP, unstable angina pectoris; PTE, pulmonary thromboembolism; AD, aortic dissection; NSTEMI, non-ST-segment elevation myocardial infarction; STEMI, ST-segment elevation myocardial infarction. **(B)** Area under the curve (AUC) for S100A12, high-sensitivity cardiac troponin T (hscTnT), creatine kinase MB isoenzyme (CK-MB) in STEMI patients. *P < 0.05 compared with all other groups.

Among the first cohort of 1,023 consecutive patients presenting with acute chest pain, 438 (42.8%) were adjudicated as STEMI. The final diagnosis in the other 585 patients were NSTEMI (*n* = 248), UAP (*n* = 88), PTE (*n* = 8), AD (*n* = 10) and “other” (*n* = 231). Baseline characteristics of STEMI versus not STEMI patients are shown in Table 1. Mean plasma concentration of S100A12 at the time of arrival in the emergency department was significantly higher in patients with STEMI (520.1 ± 301.0 ng/ml) compared with other final diagnoses (NSTEMI 205.51 ± 98.15 ng/ml; UAP 181.49 ± 65.41ng/ml; AD 170.4 ± 63.5 ng/ml; PTE 135.2 ± 45.6 ng/ml; Others 53.81 ± 26.74 ng/ml; *P* < 0.001 for all comparisons vs. STEMI; Figure 1A). By ROC analysis, at an optimal cutoff value of 202.2 ng/ml, the AUC of S100A12 for the diagnosis of STEMI was 0.969 (95%CI 0.959–0.978), which was significantly higher than the AUC for hscTnT (0.896, 95%CI 0.872–0.920) and CK-MB (0.798, 95%CI 0.767–0.829) (both *P* < 0.05; Figure 1B). The sensitivity and specificity of S100A12 for STEMI were 88.4% (95%CI 0.849–0.911) and 92.5% (95%CI 0.889–0.941) respectively, higher than that for hscTnT (sensitivity 84.2%, 95%CI 0.804–0.875, specificity 85.6%, 95%CI 0.811–0.890) and CK-MB (sensitivity 57.3%, 95%CI 0.525–0.619, specificity: 91.0%, 95%CI: 0.872–0.937).

**Table 1:** Baseline characteristics of the cohort 1 study population.

**Variable**	**Not STEMI (*n =* 585)**	**STEMI (*n =* 438)**	***P*-value**
Sex, male	400 (68.3)	354 (80.8)	<0.0001
Age, years	60.3 ± 10.5	59.0 ± 12.1	0.06
Body mass index, kg/m^2^	25.7 ± 3.3	25.6± 2.8	0.58
Heart rate, bpm	78.3 ± 12.8	79.9 ± 15.8	0.08
Blood pressure, mmHg	122.9 ± 19.5	123.5 ± 19.9	0.66
Smoking	316 (54.0)	260 (59.4)	0.09
Hypertension	339 (57.9)	252 (57.5)	0.89
Diabetes	174 (29.7)	136 (31.5)	0.65
Prior MI	92 (15.7)	35 (7.9)	0.0002
Previous stroke	98 (16.8)	82 (18.7)	0.41
Symptom onset to hospital arrival, hrs	6.4 ± 5.2	5.9 ± 5.5	0.15
TG, mmol/dl	2.0 ± 1.7	1.9 ± 1.9	0.17
HDL-C, mmol/dl	1.1 ± 0.4	1.1 ± 0.2	0.59
LDL-C, mmol/dl	2.3 ± 0.9	3.1 ± 0.8	<0.0001
GLU, mmol/dl	6.6 ± 2.2	7.7 ± 2.7	<0.0001
WBC, x 10^9^/L	7.8 ± 2.5	11.6 ± 3.9	<0.0001
Hs-CRP, mg/l	2.9 ± 4.6	5.4 ± 9.4	<0.0001
hscTnT, ng/ml	0.2 ± 0.5	0.9 ± 1.8	<0.0001
CK-MB, U/L	27.3 ± 28.9	56.0 ± 81.7	<0.0001
S100A12, ng/ml	140.4 ± 100.7	520.1 ± 301.0	<0.0001

*Data presented are means ± SD or n (%). MI, myocardial infarction; TG, triglyceride; HDL-C, high density lipoprotein; LDL-C, low-density lipoprotein; GLU, blood glucose; WBC, white blood cell; hs-CRP, high-sensitivity C-reactive protein; hscTnT, high-sensitivity troponin T; CK-MB, creatine kinase MB isoenzyme*.

**Figure 2:**
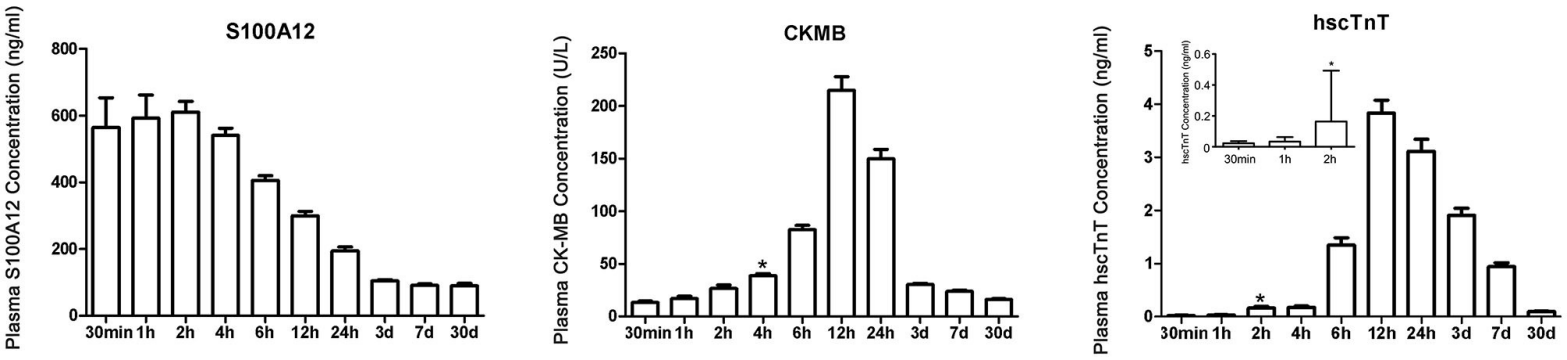
Time trends of plasma S100A12, CK-MB and hscTnT levels in patients with STEMI. *Represents the time point when plasma concentration was first higher than the diagnostic critical value. Chest pain time ≤ 30 min included 9 STEMI patients (a total of 9 samples were measured); chest pain time of 30 min to 1 h included 28 STEMI patients (a total of 37 samples were measured); and chest pain time of 1–2 h included 113 STEMI patients (a total of 150 samples were measured). Samples from all 150 patients were tested at each subsequent time after 2 h. Bars and limit lines represent the mean ± SEM.

**Figure 3:**
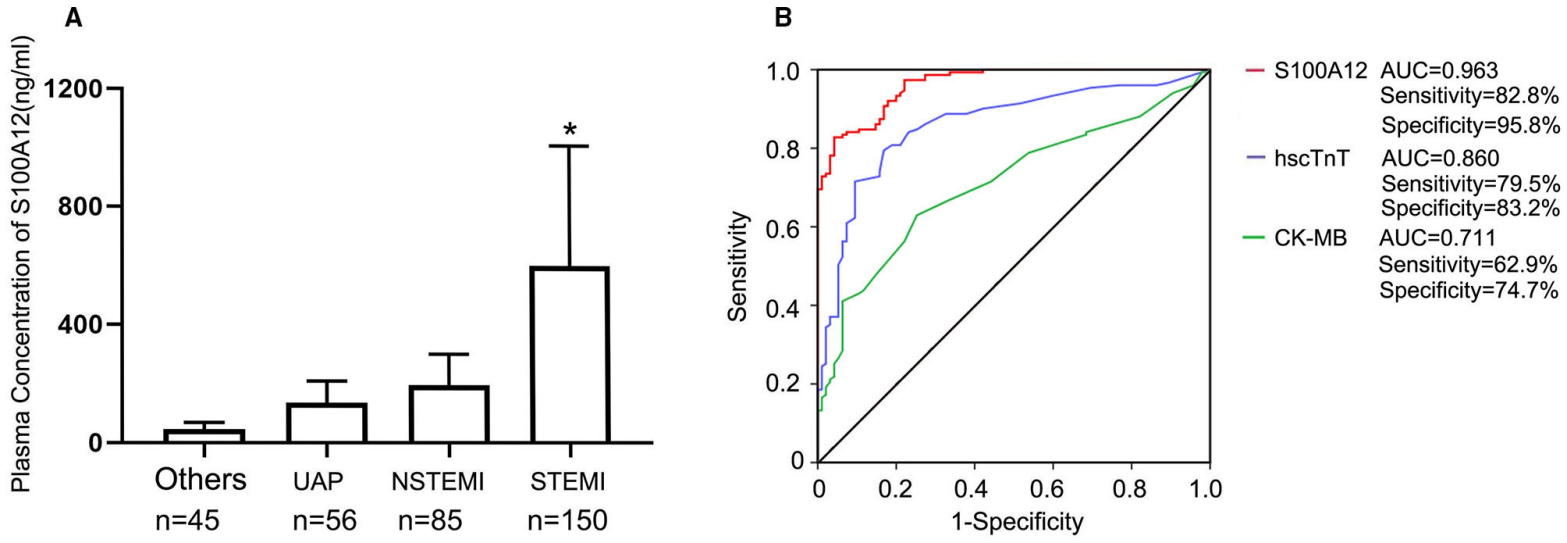
Diagnostic accuracy of S100A12 in patients presenting within 2 h of symptom onset; **(A)** Plasma concentration of S100A12 in patients with a final adjudicated diagnosis of STEMI, NSTMI, UAP and others; **(B)** The AUC for S100A12, hscTnT, and CK-MB in the STEMI cohort presenting ≤ 2 h after symptom onset (*n* = 150). UAP, unstable angina pectoris; PTE, pulmonary thromboembolism; AD, aortic dissection; NSTEMI, non-ST-segment elevation myocardial infarction; STEMI, ST-segment elevation myocardial infarction.

The time trend of plasma S100A12 levels in STEMI are shown in Figure 2. Plasma S100A12 levels were markedly elevated at 30 min after hospital arrival (the approximate time of first draw), peaked at 1–2 h and remained elevated for 12 h, followed by a gradual decline thereafter. Elevation of plasma concentrations of hscTnT and CK-MB in STEMI occurred later than S100A12 (hscTnT elevation began at 2 h and peaked at 12 h; CK-MB elevation began at 4 h and peaked at 12 h).For patients presenting with chest pain within 2 h of symptom onset (*n* = 150), plasma S100A12 levels at admission were markedly elevated in patients with a final diagnosis of STEMI (604.8 ± 441.2 ng/ml) compared with other diagnoses (143.1 ± 102.4 ng/ml) (Figure 3A). The AUC for S100A12 for the early (≤ 2 h) diagnosis of STEMI was 0.963 (95%CI 0.945–0.982), with sensitivity 82.8% (95%CI 76.2–88.7%) and specificity 95.8% (95%CI 88.9–98.6%) compared with an AUC of 0.860 for hscTnT (95%CI 0.812–0.908) with sensitivity 79.5% (95%CI 71.8–85.3%) and specificity 83.2% (95%CI 73.8–89.8%) and an AUC of 0.711 for CK-MB (95%CI 0.647–0.775) with sensitivity 62.9% (95%CI 55.0–70.9%) and specificity 74.7% (95%CI: 64.6–82.8%) (AUC for S100A12 vs. hscTnT and vs. for CK-MB both *P* < 0.05; Figure 3B).

Among the 243 patients with chest pain in the second (validation) cohort, 150 (61.7%) were adjudicated as STEMI and 93 (38.3%) were not STEMI. The baseline characteristics of the second cohort were similar to those of the first cohort (Supplementary Table S1). Plasma S100A12 levels and their diagnostic value for STEMI were consistent to those of the first cohort (Supplementary Table S2).

Patients in the third cohort (all of whom had STEMI) were stratified according to baseline tertile of plasma S100A12 levels (Table 2). Diabetes, elevated hscTnT and symptom onset time were more common in patients with high plasma S100A12 levels; other baseline characteristics were unrelated to S100A12. The 1,000 STEMI patients were initially treated with emergent primary PCI (*n* = 877 [87.7%]), fibrinolytic therapy (*n* = 43 [4.3%]), or conservatively without rapid reperfusion (*n* = 80 [8.0%]). During 1-year clinical follow-up, a total of 62 MACCE events occurred in 60 patients (6.0%). The 1-year rate of MACCE was greatest in patients in the highest peak S100A12 tertile, intermediate in the middle tertile and least in the lowest tertile (9.3 vs. 5.7 vs. 3.0% respectively, P_trend_ = 0.0006). Higher peak plasma S100A12 levels were also associated with an increased risk of all-cause death (4.2 vs. 2.4 vs. 0.9%, P_trend_ = 0.03) (Table 3). By univariable analysis advanced age, male sex, Killip class, diabetes, history of stroke, treatment with angiotensin-converting enzyme inhibitors (ACEI) and statins at discharge, peak S100A12 level were associated with 1-year MACCE. By multivariable analysis, the peak plasma S100A12 level was an independent predictor of 1-year MACCE (HR 1.001, 95%CI 1.000–1.002; *P* = 0.01) for all patients with STEMI (Table 4) and those treated with primary PCI (HR 1.001, 95%CI 1.000–1.002, *P* = 0.01; Supplementary Table S3). Peak levels of hscTnT and CK-MB were not significantly associated with 1-year MACCE.

**Table 2:** Baseline characteristics of cohort 3 STEMI patients stratified by baseline S100A12 tertiles.

**Variable**	**Overall (*n =* 1,000)**	**Plasma S100A12 levels on admission**			
		**Lowest tertile (*n =* 333)**	**Median tertile (*n =* 332)**	**Highest tertile (*n =* 335)**	***P*-value**
Sex, male	811 (81.1)	272 (81.7)	272 (81.9)	267 (79.7)	0.72
Age, years	59.4 ± 12.1	60 ± 12.6	59 ± 11.2	59.3 ± 12.5	0.59
Hypertension	597 (59.7)	198 (59.5)	209 (62.9)	190 (56.7)	0.26
Current smoking	572 (57.2)	187 (56.2)	180 (54.2)	205 (61.2)	0.17
Diabetes	336 (33.6)	96 (28.8)	107 (32.2)	133 (39.7)	0.01
Prior MI	89 (8.9)	30 (9.0)	22 (6.7)	37 (11.0)	0.13
Previous stroke	186 (18.6)	62 (18.6)	59 (17.8)	65 (19.4)	0.86
Symptom onset to hospital arrival, hrs	6.5 ± 5.0	8.2 ± 6.3	6.1 ± 4.2	5.4 ± 3.8	<0.0001
Killip class ≥2	200 (2.0)	57 (17.1)	72 (21.7)	71 (21.2)	0.27
Primary PCI	877 (87.7)	292 (87.7)	293 (88.3)	292 (87.2)	0.91
Thrombolysis	43 (4.3)	15 (4.5)	15 (4.5)	13 (3.8)	0.89
No reperfusion	80 (8.0)	24 (7.2)	25 (7.5)	31 (9.3)	0.58
Radial access	834 (83.4)	275 (82.6)	276 (83.1)	283 (84.5)	0.78
Femoral access	134 (13.4)	48 (14.4)	44 (13.3)	42 (12.5)	0.78
**Infarct artery**
LAD	650 (67.1)	214 (66.3)	223 (69.7)	213 (65.5)	0.49
LCX	279 (28.8)	97 (30.0)	89 (27.8)	93 (28.6)	0.82
RCA	457 (47.2)	160 (49.5)	142 (44.4)	155 (47.7)	0.41
LM	17 (1.8)	3 (0.9)	8 (2.5)	6 (1.8)	0.31
Use of Bivalirudin	286 (29.5)	82 (25.4)	95 (29.7)	109 (33.5)	0.08
LVEF, % (mean ± SD)	56 ± 8	56 ± 9	55 ± 9	55 ± 8	0.43
Aspirin	991 (99.1)	330 (99.4)	329 (99.1)	332 (99.1)	1.00
Clopidogrel/Ticagrelor	993 (99.3)	330 (99.4)	331 (99.7)	331 (98.8)	0.61
β-Blockers	814 (81.4)	273 (81.9)	267 (80.4)	274 (81.8)	0.85
ACE inhibitors	742 (74.2)	242 (72.6)	253 (76.2)	247 (73.7)	0.57
Statins	966 (96.6)	320 (96.1)	322 (96.9)	324 (96.7)	0.81
TG, mmol/dl	1.8 ± 1.8	1.7 ± 1.9	1.9 ± 1.9	1.8 ± 1.7	0.69
LDL-C, mmol/dl	3.1 ± 0.8	3.0 ± 0.8	3.1 ± 0.8	3.1 ± 0.9	0.37
HDL-C, mmol/dl	1.0 ± 0.2	1.0 ± 0.2	1.0 ± 0.2	1.1 ± 0.2	0.28
GLU, mmol/dl	8.1 ± 3.0	7.9 ± 3.0	8.1 ± 2.8	8.2 ± 3.3	0.43
WBC, 10^9^/L	11.5 ± 3.9	11.4 ± 3.8	11.4 ± 3.9	11.8 ± 4.0	0.26
hscTnT, ng/ml	0.9 ± 1.8	0.60 ± 1.5	0.9 ± 2.0	0.9 ± 1.9	0.01
Hs-CRP, mg/l	5.7 ± 9.1	5.7 ± 9.3	5.8 ± 9.4	5.5 ± 8.4	0.91
CK-MB, U/L	56.8 ± 79.9	54.8 ± 84.6	57.9 ± 77.2	57.6 ± 78.2	0.87
S100A12, ng/ml	517.7 ± 306.7	206.9 ± 72.7	498.9 ± 116.4	845.3 ± 233.7	<0.0001

*Data are mean ± SD or n (%). PCI, percutaneous coronary intervention; LAD, left anterior descending; LCX, left circumflex artery; RCA, right coronary artery; LM, left main; LVEF, left ventricular ejection fraction; ACE, angiotensin-converting enzyme; MI, myocardial infarction; TG, triglyceride; HDL-C, high density lipoprotein; LDL-C, low-density lipoprotein; GLU, blood glucose; WBC, white blood cell; hs-CRP, high-sensitivity C-reactive protein; hscTnT, high-sensitivity troponin T; CK-MB, creatine kinase MB isoenzyme*.

**Table 3:** One-year clinical outcomes in cohort 3 STEMI patients according to peak S100A12 tertiles.

**Endpoint**	**Lowest tertile (*n =* 333)**	**Middle tertile (*n =* 332)**	**Highest tertile (*n =* 335)**	**Trend *P*-value**
MACCE^*^	10 (3.0%)	19 (5.7%)	31 (9.3%)	0.0006
- All-cause death^*^	5 (0.9%)	8 (2.4%)	14 (4.2%)	0.03
- Reinfarction	1 (0.3%)	4 (1.2%)	5 (1.8%)	0.12
- Stroke	2 (0.6%)	2 (0.6%)	6 (1.8%)	0.12
- Heart failure hospitalization	2 (0.6%)	6 (1.8%)	7 (2.1%)	0.12

*MACCE, major adverse cardiac and cerebral events.*

* *P < 0.05 between three groups*.

**Table 4:** Independent predictors of 1-year MACCE in cohort 3 patients with STEMI.

**Variable**	**Multiple regression**	
	**HR (95% CI)**	***P*-value**
Age, years	1.009 (0.986-1.034)	0.45
Sex, male	0.661 (0.366–1.193)	0.17
Diabetes	1.748 (1.028–2.971)	0.04
Previous stroke	1.772 (1.007–3.120)	0.047
Killip class ≥2	2.214 (1.273–3.851)	0.005
Primary PCI	0.728 (0.368–1.443)	0.36
ACE inhibitor use at discharge	0.513 (0.298–0.882)	0.02
Statin use at discharge	0.282 (0.108–0.737)	0.01
Symptom onset to hospital arrival, hrs	1.019 (0.968–1.073)	0.47
Peak hsTnT, ng/ml	1.009 (0.923–1.023)	0.84
Peak CK–MB, U/L	1.001 (0.999–1.002)	0.39
Peak S100A12, ng/ml	1.001 (1.000–1.002)	0.01

*PCI, percutaneous coronary intervention; LAD, left anterior descending; LCX, left circumflex artery; RCA, right coronary artery; LM, left main; LVEF, left ventricular ejection fraction; ACE, angiotensin-converting enzyme; MI, myocardial infarction; TG, triglyceride; HDL-C, high density lipoprotein; LDL-C, low-density lipoprotein; GLU, blood glucose; WBC, white blood cell; hs-CRP, high-sensitivity C-reactive protein; hscTnT, high-sensitivity troponin T; CK-MB, creatine kinase MB isoenzyme*.

## Discussion

The present study is the first investigation to our knowledge to evaluate the diagnostic accuracy and prognostic utility of plasma S100A12 levels in patients with chest pain and confirmed STEMI. The major findings are as follows: First, a high plasma level of S100A12 was identified in patients with STEMI, the occurrence of which provided higher diagnostic and differential diagnostic accuracy than hscTnT and CK-MB levels. Second, plasma S100A12 levels were elevated at a very early stage of STEMI (beginning at 30 min and peaking at 1–2 h), significantly earlier than other classic myocardial biomarkers, providing higher sensitivity and specificity for the diagnosis of STEMI within 2 hours of symptom onset. Third, S100A12 expression was strong in ruptured plaques and coronary artery thrombi of patients with STEMI, co-localizing in CD68+ macrophages, and was not elevated after myocardial necrosis from other causes. Finally, the peak plasma S100A12 level was an independent predictor of MACCE at 1-year in patients with STEMI, indicating its potential clinical utility for risk stratification.

S100A12 is endogenously expressed by cells closely linked to vascular disease including granulocytes, myeloid cells and macrophages (14, 15). Consistent with the findings from previous studies, the immunohistochemistry and immunofluorescence findings from the present study in acutely aspirated coronary thrombi and autopsy specimens from patients with STEMI suggests that the increase in plasma S100A12 in STEMI derives from its acute release from CD68+ macrophages in ruptured plaques, a mechanism quite different than the elevation of biomarkers derived from myocardial necrosis such as CK-MB and hscTnT (16). Plasma S100A12 was slightly increased in patients with non-plaque related chest pain, such as PTE and AD, possibly due to systemic or focal inflammation. However, extremely high levels of S100A12 were only seen with STEMI. We also observed that plasma S100A12 levels were low in patients with stable CAD, slightly increased in UAP and modestly increased in NSTEMI. This suggests that plasma S100A12 levels might reflect plaque burden, composition, focal inflammation, and instability of thrombotic atherosclerotic lesions. In this regard optical coherence tomography studies have reported that plaque ruptures occurred in 71% of patients presenting with sudden death after STEMI vs. 43% after NSTEMI (17). It has further been described that plaques in STEMI are usually more severe, larger and have greater lipid and macrophage content compared with those in NSTEMI. These distinctions may underlie the differences in plasma S100A12 levels between STEMI and NSTEMI.

A notable implication from the present study regards the potential utility of S100A12 for the early diagnosis of STEMI. Elevation of classic myocardial biomarkers such as hscTnT and CK-MB do not occur for 2–4 h after onset of STEMI as seen in the present and previous studies (18, 19). This delay may lead to uncertainty in the rapid diagnosis of STEMI, especially in patients presenting early with atypical symptoms and/or ECGs. In patients with STEMI the elevation of S100A12 due to coronary artery plaque rupture and thrombi formation precedes the rise in hscTnT and CK-MB due to subsequent myonecrosis. Plasma S100A12 was elevated as soon as 30 min after STEMI onset and peaked at 1–2 h, providing very good sensitivity and excellent specificity for the differential diagnosis of symptoms within 2 h, substantially better than for hscTnT and CK-MB. The earlier and more accurate discrimination of STEMI provided by S100A12 may afford faster triage and initiation of reperfusion therapy, potentially improving clinical outcomes.

In addition to its value in the early diagnosis of STEMI, the peak level of S100A12 was an independent predictor of the 1-year prognosis after STEMI, in particular all-cause mortality. Although the precise mechanisms are unknown, several possible reasons might explain the association between S100A12 and clinical outcomes. First, inflammation plays a central role in the development and progression of atherosclerosis. Previous studies have demonstrated that hs-CRP, a classic biomarker of systemic inflammation, is useful in predicting adverse clinical outcomes in various cardiovascular disease settings (20, 21). Like hs-CRP, S100A12 is a sensitive concentration-dependent biomarker which correlates with the extent of inflammation and outcomes, as demonstrated in the present and previous studies (22, 23). Notably, in the present study the level of S100A12 was a stronger predictor of prognosis than was hs-CRP. Second, as S100A12 is derived from ruptured coronary plaques and is highly expressed in coronary thrombi, its concentration may reflect the severity of the underlying atherosclerotic plaque (ruptured and stable), as an indicator of the vulnerability of the patient. Notably, the peak levels of hscTnT and CK-MB were not associated with MACCE at 1-year in the present study.

Our study has limitations. First, although the principal findings from the diagnosis cohort were validated in a second dataset from 3 independent hospitals, additional confirmatory studies should be performed in an even larger population and in different geographies. Second, plasma S100A12 concentrations currently must be measured by ELISA, and commercial clinical laboratory analyzers such as those widely used for hscTnT and CK-MB are unavailable. Third, S100A12 is not specific to cardiac disease. Other diseases such as stroke and immunologic and infectious diseases are also associated with elevation of plasma S100A12 levels. Thus, the diagnostic accuracy of S100A12 to diagnose STEMI will depend on the clinical characteristics and presentation of the test population; like hscTnT, its specificity will likely decline if applied to non-cardiac and miscellaneous conditions. Finally, we cannot rule out the role of unmeasured confounders in our multivariable model.

In conclusion, in the present large-scale study, plasma levels of S100A12 were elevated in the early stage of STEMI, had stronger discrimination for the diagnosis of STEMI than CK-MB or hscTnT, and were an independent predictor of 1-year MACCE, including all-cause mortality. Compared to classic biomarkers of myocardial necrosis, S100A12 may offer several advantages for the early evaluation of patients with chest pain to guide rapid reperfusion therapy and provide prognostic utility in patients with STEMI.

## Data Availability Statement

The original contributions presented in the study are included in the article/Supplementary Material, further inquiries can be directed to the corresponding author/s.

## Ethics Statement

The studies involving human participants were reviewed and approved by Human Ethics Committee of the General Hospital of Northern Theater Command. The patients/participants provided their written informed consent to participate in this study. The animal study was reviewed and approved by Human Ethics Committee of The General Hospital of Northern Theater Command.

## Author Contributions

YH was responsible for the study concept and design and obtained study funding. YH, MC, XZ, and YL wrote the manuscript. XW, BL, JD, SW, HT, and GW provided clinical specimens. MC, XZ, NG, YL, and MQ did the statistical analysis. XM and GS were responsible for major revision of the manuscript. All authors critically revised the paper and approved the final version.

## Funding

This work was supported by grants from the National Key Research and Development project of China [grant number 2016YFC1301300]. The funders of the study had no role in study design, data collection, analysis, interpretation, or writing of the report. The authors had access to the data and vouch for the integrity, accuracy, and completeness of the data and analyses, and for the fidelity of the study to the protocol.

## Conflict of Interest

GS has served as a consultant to Claret, Backbeat, Sirtex, Matrizyme, Miracor, Neovasc, V-wave, Shockwave, Valfix, TherOx, Reva, Vascular Dynamics, Robocath, HeartFlow, Gore, Ablative Solutions, MAIA Pharmaceuticals, Abiomed and Ancora; has received speaker honoraria from Terumo and Amaranth; has equity/options in Ancora, Cagent, Qool Therapeutics, Aria, MedFocus family of funds, Biostar family of funds, Applied Therapeutics and SpectraWAVE; is a director of SpectraWave; and his employer, Columbia University, receives royalties for sale of the MitraClip from Abbott. The remaining authors declare that the research was conducted in the absence of any commercial or financial relationships that could be construed as a potential conflict of interest.

## Publisher's Note

All claims expressed in this article are solely those of the authors and do not necessarily represent those of their affiliated organizations, or those of the publisher, the editors and the reviewers. Any product that may be evaluated in this article, or claim that may be made by its manufacturer, is not guaranteed or endorsed by the publisher.

## Abbreviations

AMI: acute myocardial infarction
AUC: area under the curve
CK-MB: creatine kinase isoenzyme
hscTnT: high-sensitivity cardiac troponin T
MACCE: major adverse cardiac and cerebral events
NSTEMI: non-ST-segment elevated myocardial infarction
STEMI: ST-segment elevation myocardial infarction
UAP: unstable angina pectoris.
