# Corrigendum: Utility of S100A12 as an Early Biomarker in Patients With ST-Segment Elevation Myocardial Infarction

**DOI:** 10.3389/fcvm.2022.840102

**Published:** 2022-02-23

**Authors:** Xiaolin Zhang, Minghui Cheng, Naijing Gao, Yi Li, Chenghui Yan, Xiaoxiang Tian, Dan Liu, Miaohan Qiu, Xiaozeng Wang, Bo Luan, Jie Deng, Shouli Wang, Hongyan Tian, Geng Wang, Xinliang Ma, Gregg W. Stone, Yaling Han

**Affiliations:** ^1^Cardiovascular Research Institute and Department of Cardiology, The General Hospital of Northern Theater Command, Shenyang, China; ^2^Department of Cardiology, Liaoning Provincial People's Hospital, Shenyang, China; ^3^Department of Cardiology, Second Affiliated Hospital of Xi'an Jiaotong University, Xi'an, China; ^4^Department of Cardiology, General Hospital of the Strategic Support Force of the Chinese People's Liberation Army, Beijing, China; ^5^Department of Cardiology First Affiliated Hospital of Xi'an Jiaotong University, Xi'an, China; ^6^Department of Emergency Medicine, Thomas Jefferson University, Philadelphia, PA, United States; ^7^Icahn School of Medicine at Mount Sinai, Mount Sinai Heart and the Cardiovascular Research Foundation, New York, NY, United States

**Keywords:** S100A12, ST-segment elevation myocardial infarction, diagnosis, prognosis, cardiovascular disease(s)

In the original article, the Funding statement is incorrect. The authors neglected to include the funder “National Key Research and Development Program of China, 2016YFC1301303” for author YH and the funder “Liaoning Provincial Natural Science Foundation of China, 2019-ZD-1060” for author XZ. The corrected Funding statement appears below:

“This work was supported by grants from the National Key Research and Development project of China [grant number 2016YFC1301300]. YH received funding from the National Key Research and Development Program of China, 2016YFC1301303. XZ received funding from the Liaoning Provincial Natural Science Foundation of China, 2019-ZD-1060. The funders of the study had no role in study design, data collection, analysis, interpretation, or writing of the report. The authors had access to the data and vouch for the integrity, accuracy, and completeness of the data and analyses, and for the fidelity of the study to the protocol.”

In the original article, an error was made in the **Abstract**, section *Design, Setting, and Participants*. The original sentence was incorrectly written as: “An independent cohort of 398 patients enrolled at three different hospitals served as a validation cohort.” The correct sentence is: “An independent cohort of 243 patients enrolled at three different hospitals served as a validation cohort.”

In the original article, an error was made in the **Methods**, section *Study Design and Participants*. The original sentence was incorrectly written as: “A second patient group, serving as a validation cohort, comprised 398 patients with the same inclusion and exclusion criteria presenting to the emergency department of three different hospitals (eAppendix 1) between May 2016, and November 2016”. The correct sentence is: “A second patient group, serving as a validation cohort, comprised 243 patients with the same inclusion and exclusion criteria presenting to the emergency department of three different hospitals (eAppendix 1) between May 2016 and November 2016.”

The authors apologize for these errors and state that they do not change the scientific conclusions of the article in any way. The original article has been updated.

## Publisher's Note

All claims expressed in this article are solely those of the authors and do not necessarily represent those of their affiliated organizations, or those of the publisher, the editors and the reviewers. Any product that may be evaluated in this article, or claim that may be made by its manufacturer, is not guaranteed or endorsed by the publisher.

