# Supplementary material for: Utility of S100A12 as an Early Biomarker in Patients With ST-Segment Elevation Myocardial Infarction
Source: Front Cardiovasc Med. 2021 Dec 17;8:747511. doi: 10.3389/fcvm.2021.747511 (PMC8718434; doi:10.3389/fcvm.2021.747511)
Supplement: Supplementary file 1 [file Data_Sheet_1.docx]

**SUPPLEMENTAL MATERIAL**

**Utility of S100A12 as an Early Biomarker in Patients with ST-Segment Elevation Myocardial Infarction**

| **Supplemental Table S1.** Clinical characteristics of patients with STEMI in the first and second cohorts compared | Pg 2 |
| --- | --- |
| **Supplemental Table S2.** Diagnostic accuracy of plasma S100A12 level in the first and second cohorts | Pg 4 |
| **Supplemental Table S3.** Independent predictors of 1-year MACCE in cohort 3 patients with STEMI treated with primary PCI | Pg 5 |
| **Online Figure 1.** Flow of the Study Population | Pg 6 |
| **eAppendix 1.** Hospitals involved in the validation study | Pg 7 |
| **eAppendix 2.** Blood samples storage and biochemical measurements | Pg 7 |

**Table S1. Clinical characteristics of patients with STEMI in the first and second cohorts compared**

| **Variable** | **First cohort** | **Second cohort** | ***P* Value** |
| --- | --- | --- | --- |
|  | **STEMI (n=438)** | **STEMI (n=150)** |  |
| Sex, male | 354 (80.8) | 125 (83.3) | 0.49 |
| Age, years | 59.0 ± 12.1 | 60.1 ± 11.6 | 0.44 |
| LVEF | 56 ± 8.5 | 56 ± 8.9 | 0.81 |
| Heart rate, bpm | 79.9 ± 15.8 | 81.3 ± 12.8 | 0.26 |
| Blood pressure, mmHg | 123.5 ± 19.9 | 125.2 ± 23.7 | 0.28 |
| Current smoking | 260 (59.4) | 81 (54.0) | 0.25 |
| Hypertension | 252 (57.5) | 101 (67.3) | 0.0344 |
| Diabetes | 136 (31.5) | 57 (38.0) | 0.12 |
| Previous stroke | 82 (18.7) | 36 (24.0) | 0.16 |
| Symptom onset to hospital arrival, hrs | 5.9 ± 5.5 | 6.5 ± 4.7 | 0.15 |
| TG, mmol/dl | 1.9 ± 1.9 | 1.7 ± 1.4 | 0.28 |
| LDL-C, mmol/dl | 3.1 ± 0.8 | 3.1 ± 0.8 | 0.24 |
| GLU, mmol/dl | 7.7 ± 2.7 | 7.8 ± 2.9 | 0.78 |
| WBC, 10^9^/L | 11.6 ± 3.9 | 11.8 ± 4.3 | 0.41 |
| hscTnT, ng/ml | 0.9 ± 1.8 | 1.1 ± 2.2 | 0.35 |
| CK-MB, U/L | 56.0 ± 81.7 | 60.9 ± 82.0 | 0.62 |
| S100A12, ng/ml | 520.1 ± 301.0 | 483.9 ± 250.7 | 0.15 |

Data presented are means ± SD or n (%). TG, triglyceride; HDL-C, [high](http://cn.bing.com/dict/clientsearch?mkt=zh-CN&setLang=zh&form=BDVEHC&ClientVer=BDDTV3.5.0.4311&q=%E9%AB%98%E5%AF%86%E5%BA%A6%E8%84%82%E8%9B%8B%E7%99%BD) [density](http://cn.bing.com/dict/clientsearch?mkt=zh-CN&setLang=zh&form=BDVEHC&ClientVer=BDDTV3.5.0.4311&q=%E9%AB%98%E5%AF%86%E5%BA%A6%E8%84%82%E8%9B%8B%E7%99%BD) [lipoprotein](http://cn.bing.com/dict/clientsearch?mkt=zh-CN&setLang=zh&form=BDVEHC&ClientVer=BDDTV3.5.0.4311&q=%E9%AB%98%E5%AF%86%E5%BA%A6%E8%84%82%E8%9B%8B%E7%99%BD); LDL-C, [low](http://cn.bing.com/dict/clientsearch?mkt=zh-CN&setLang=zh&form=BDVEHC&ClientVer=BDDTV3.5.0.4311&q=%E4%BD%8E%E5%AF%86%E5%BA%A6%E8%84%82%E8%9B%8B%E7%99%BD)-[density](http://cn.bing.com/dict/clientsearch?mkt=zh-CN&setLang=zh&form=BDVEHC&ClientVer=BDDTV3.5.0.4311&q=%E4%BD%8E%E5%AF%86%E5%BA%A6%E8%84%82%E8%9B%8B%E7%99%BD) [lipoprotein](http://cn.bing.com/dict/clientsearch?mkt=zh-CN&setLang=zh&form=BDVEHC&ClientVer=BDDTV3.5.0.4311&q=%E4%BD%8E%E5%AF%86%E5%BA%A6%E8%84%82%E8%9B%8B%E7%99%BD); GLU, [blood](http://cn.bing.com/dict/clientsearch?mkt=zh-CN&setLang=zh&form=BDVEHC&ClientVer=BDDTV3.5.0.4311&q=%E8%A1%80%E7%B3%96) [glucose](http://cn.bing.com/dict/clientsearch?mkt=zh-CN&setLang=zh&form=BDVEHC&ClientVer=BDDTV3.5.0.4311&q=%E8%A1%80%E7%B3%96); WBC, white blood cell; hs-CRP, high-sensitivity C-reactive protein; hscTnT, high-sensitivity troponin T; CK-MB, creatine kinase MB isoenzyme.

**Table S2. Diagnostic accuracy of plasma S100A12 level in the first and second cohorts**

|  | **First cohort** | **Second cohort** |
| --- | --- | --- |
|  | **STEMI (n=438)** | **STEMI (n=150)** |
| AUC | 0.969 | 0.914 |
| Sensitivity | 88.4% | 82.0% |
| Specificity | 92.5% | 86.0% |

AUC, area under the curve.

**Table S3. Independent predictors of 1-year MACCE in cohort 3 patients with STEMI treated with primary PCI**

| **Variable** | **Multiple regression** | |
| --- | --- | --- |
|  | **HR (95% CI) P value** | |
| Age, years | 1.010 (0.984-1.038) | 0.45 |
| Sex, male | 0.775 (0.392-1.530) | 0.46 |
| Diabetes | 2.050 (1.143-3.667) | 0.0159 |
| Previous stroke | 1.626 (0.846-3.126) | 0.15 |
| Killip class ≥2 | 2.233 (1.201-4.152) | 0.0110 |
| Symptom onset to hospital arrival, hrs | 0.994 (0.929-1.063) | 0.85 |
| ACE inhibitor use | 0.382 (0.211-0.690) | 0.0014 |
| Statin use | 0.339 (0.100-1.150) | 0.08 |
| Peak hsTnT, ng/ml | 1.026 (0.932-1.130) | 0.59 |
| Peak CK-MB, U/L | 1.001 (1.000-1.002) | 0.17 |
| Peak S100A12 tertile* | 1.001 (1.000-1.002) | 0.0154 |

Abbreviations per Table 2.

**Online Figure 1.** Flow of the Study Population

**
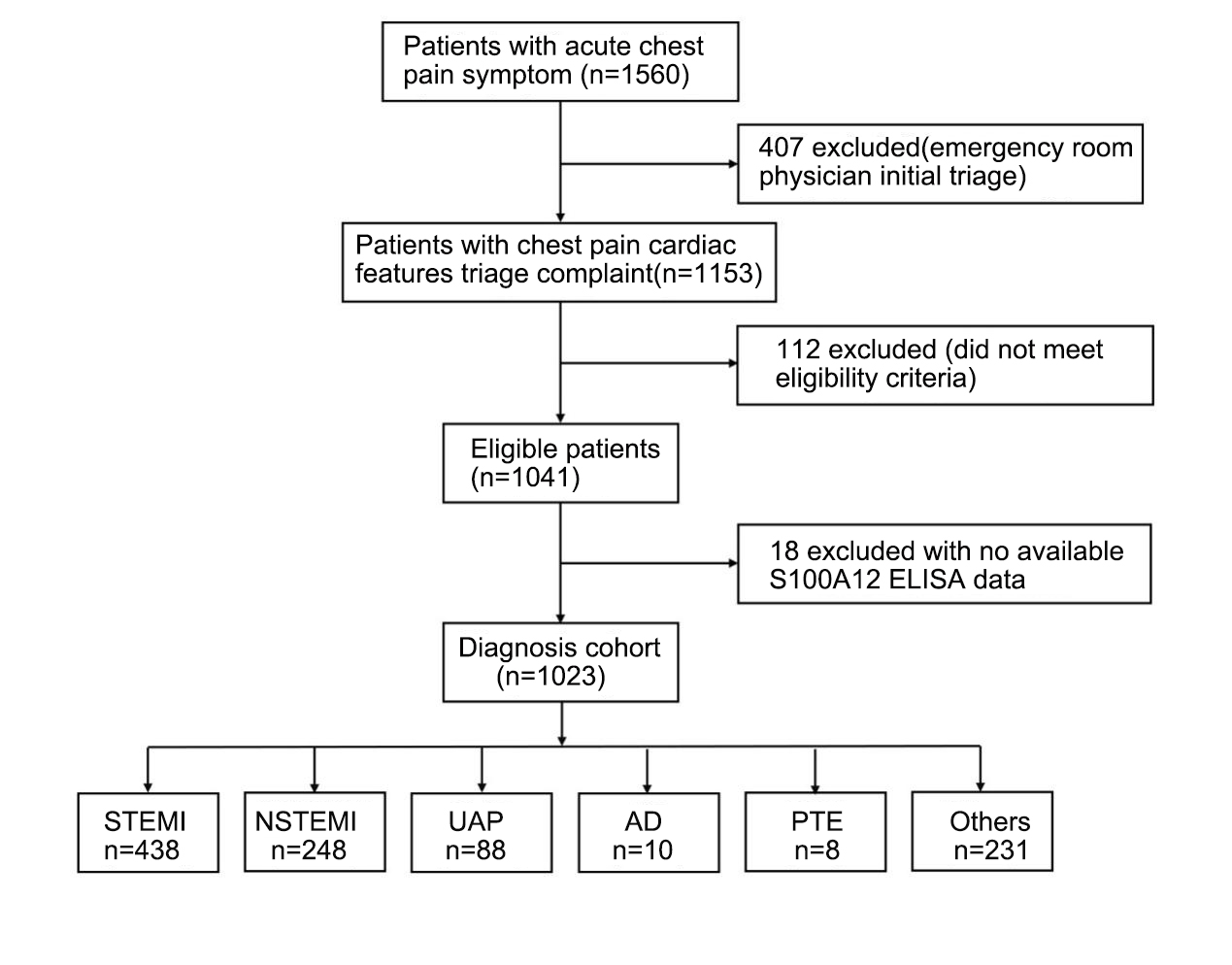
**

**eAppendix 1.** Hospitals involved in the validation study

1.People's Hospital of Liaoning Province, Shenyang;

2. Second Affiliated Hospital of Xi'an Jiao Tong University, Xi’an;

3.The General Hospital of the strategic Support Force of the Chinese People’s Liberation Army, Beijing.

**eAppendix 2. Blood samples storage and biochemical measurements**

Blood samples were collected in EDTA-anticoagulation tubes and plasma was isolated by centrifuging at 4°C for 15m at 1000 × g. Samples were stored in plastic cryovials at -80°C or colder. Testing for hscTnT and CK-MB were performed by personnel blinded to clinical diagnosis. Plasma concentrations of S100A12 were determined using an enzyme-linked immunosorbent assay (ELISA) according to the manufacturer’s instructions (CircuLex S100A12/EN-RAGE ELISA kit, CycLex, Nagano, Japan).
